# Prevalence of insulin dysregulation in a sport horse population as determined by traditional and stall-side testing

**DOI:** 10.1002/evj.70204

**Published:** 2026-07-01

**Authors:** Kimberly L. Hallowell, Caitlyn R. Horne, Cary S. Miller, Sara R. Tufts, Katarzyna Dembek, Kim R. Love, Andrew W. van Eps, Lauren V. Schnabel

**Affiliations:** 1 Department of Clinical Sciences, North Carolina State University College of Veterinary Medicine, Raleigh, North Carolina, USA; 2 K. R. Love Quantitative Consulting and Collaboration, Athens, Georgia, USA; 3 Department of Clinical Studies, New Bolton Center, School of Veterinary Medicine, University of Pennsylvania, Kennett Square, Pennsylvania, USA; 4 Comparative Medicine Institute, North Carolina State University, Raleigh, North Carolina, USA

**Keywords:** endocrine, equine metabolic syndrome, horse, hyperinsulinemia, lateral flow assay

## Abstract

**Background:**

The prevalence of insulin dysregulation (ID) has not been investigated in sport horses. Stall-side insulin assays offer convenience but need further validation.

**Objectives:**

To determine the prevalence of and risk factors for ID in a sport horse population using an oral sugar test (OST) and assess agreement between stall-side and validated laboratory assays for insulin quantification.

**Study Design:**

Prospective cross-sectional study.

**Methods:**

One hundred eighty sport horses presenting for orthopaedic evaluation at a university hospital (*N* = 140) or local farms (*N* = 40) were tested for ID using OST (0.15 mL/kg light corn syrup). Morphometric data were collected, and baseline serum leptin and adiponectin concentrations were measured. Insulin concentrations were measured using the validated fluorescence enzyme immunoassay (FEIA) and a stall-side lateral flow assay (LFA). ID was diagnosed if FEIA insulin measured >20 μIU/mL at T0 or >30 μIU/mL at T60/T90. Factors associated with ID were assessed using a mixed effects binary logistic regression model. Assay agreement was assessed using Pearson’s correlation coefficient and Bland–Altman plot. Receiver operator characteristic curve analysis was performed to determine diagnostic cut-offs for LFA.

**Results:**

ID was diagnosed in 22.8% (41/180) of horses. Only 11/41 ID horses had increased fasted insulin. Increasing leptin/adiponectin ratio in males was the only significant predictor of ID (OR = 1.102, 95% CI 1.036–1.171). Correlation between FEIA and LFA insulin measures was good (*r* = 0.81, 95% CI 0.78–0.84), but the LFA read on average 13.62 μIU/mL higher than the FEIA and had greater variability at higher insulin concentrations. Area under the ROC curve showed the LFA was excellent at correctly classifying ID status (AUC = 0.95, 95% CI 0.93–0.97).

**Main Limitations:**

Single-centre study, voluntary enrolment may overestimate ID prevalence.

**Conclusions:**

ID is common in sport horses and often requires dynamic insulin testing to diagnose. Physical appearance does not predict ID risk. Agreement between stall-side and laboratory assays is good.

## 1 | INTRODUCTION

Insulin dysregulation (ID) is the hallmark of Equine Metabolic Syndrome and has been identified as a driving factor in laminitis development.^1–3^ Recent literature has highlighted the risk of hyperinsulinemia following intraarticular treatment with corticosteroids, the severity of which is increased in horses with basal hyperinsulinemia.^4–7^ These findings are of particular concern in sport horses, in which joint therapy with corticosteroids is common.

Diagnosis of ID relies on identification of basal hyperinsulinemia, hyperinsulinemia in response to ingested non-structural carbohydrates, or identification of tissue insulin resistance. Basal insulin concentrations >20 μIU/mL measured via chemiluminescent assay have good specificity but poor sensitivity for the diagnosis of ID.^8^ Diagnostic sensitivity can be improved with an oral sugar test (OST) which assesses the insulin response to light corn syrup administered by mouth.^9^ The two published studies regarding the prevalence of ID in the United States rely on different testing methods and examine disparate populations of horses. Pleasant et al. found a prevalence of 18.3% in 300 light breed horses, but relied on basal insulin testing for diagnosis.^10^ Heaton et al. found a prevalence of only 4.8% using an OST in 62 stock-type horses, but specifically tested non-obese animals.^11^ Given that 45% of the equine population is overweight or obese, and increased adiposity is a known risk factor for ID, both of these studies likely underestimate the true prevalence of ID.^12–15^

Although the OST is considered superior to basal insulin testing,^16^ waiting 60–90 min for samples can be burdensome for practitioners. Additionally, sending samples to an outside laboratory can result in delays of several days before results are available. The production of stall-side insulin assays has provided practitioners with a tool that can improve the practicality of testing. With these assays, practitioners can assess patients for ID to make management modifications immediately, saving time and reducing client costs. The Well-ness Ready (Wellness Ready, Kentucky, USA) 15-min lateral flow assay has been validated in a laboratory setting,^17^ but requires further field validation and the determination of diagnostic cut-offs. Biomarkers that can be measured at a single time point such as leptin, adiponectin, and their ratio have shown utility for differentiating ID and non-ID horses, or for predicting future laminitis risk, but require investigation in broader horse populations.^1,2,18^

The aim of this study was to determine the prevalence of insulin dysregulation in a population of sport horses of mixed breeds and disciplines using an OST and to identify factors associated with disease risk. Secondarily, we aimed to assess agreement between a stall-side assay (Wellness Ready) and a previously validated laboratory assay and to establish diagnostic cut-offs for the stall-side assay. We hypothesised that the prevalence of insulin dysregulation would be higher than the previously reported 18.3%, and that agreement between the stall-side and laboratory assays would be good.

## 2 | MATERIALS AND METHODS

### 2.1 | Animals

One hundred eighty horses and ponies aged ≥2 years were prospectively enrolled, including 140 recruited during visits to the Equine Orthopaedic Surgery and Sports Medicine Service at the North Carolina State University Equine Hospital, and 40 recruited from performance horse farms in North Carolina. Horses were eligible for enrolment if they had no prior history of veterinarian-diagnosed equine metabolic syndrome or laminitis. Horses recruited from the hospital population included those presenting for poor performance, lameness evaluation, advanced diagnostic imaging, and elective orthopaedic surgical procedures. Enrolment was offered via email at time of appointment scheduling, and if no response to the email enrolment form was obtained, enrolment was again offered by the clinical team overseeing the patient’s care at time of patient arrival. Enrolment was not offered to severely lame (grade 4 or 5) horses, horses with other sources of severe pain, or horses with systemic illness to limit the potential impact of these conditions on insulin dynamics. Local performance farms willing to participate in the study were identified via communication with referring veterinarians and trainers. Explicit informed consent was obtained from all owners prior to testing. The consent form included ‘yes’ or ‘no’ questions regarding the horse’s history of veterinarian-diagnosed equine metabolic syndrome, laminitis, or pituitary pars intermedia dysfunction (PPID). Owners were asked to report any current systemic medications the horse was receiving but were not asked about recent intraarticular or topical therapies. As part of study enrolment, foot balance radiographs of all four feet were obtained in addition to the endocrine testing described here. Owners were provided the results of stall-side insulin testing as well as JPEG images of the foot balance radiographs as incentive for study enrolment.

All animals were evaluated between February 2024 and August 2025. Horses in the hospital population were allowed to acclimate to the hospital for at least 3 h prior to evaluation. Horses in the field population were examined in their normal environment. Following routine physical examination, body condition score (BCS, 1–9),^19^ cresty neck score (CNS, 0–5),^20^ and body condition index (BCI)^21^ values were recorded. For the hospital population, weight was determined using a digital scale, and for the field population, weight was determined using a weight tape designed for horses. All assessments were performed by the same veterinarian to limit variability in scoring.

### 2.2 | Endocrine testing

All horses were fasted from grain for >8 h and from hay and pasture for 3–6 h.^16^ For the hospital population, if horses were to be tested the day of arrival to the hospital, owners were instructed to withhold morning grain. Verbal confirmation that these instructions were followed was obtained by the clinical team upon patient arrival. Fasting from hay was performed by the investigators during the hospital acclimation period, and clinical evaluation including lameness examination was delayed until the completion of the OST. Most horses in the hospital population were presenting for procedures requiring overnight hospitalisation (magnetic resonance imaging, nuclear scintigraphy, surgical procedures) and were tested the morning after arrival. In this case, all fasting was performed by the investigators in hospital, and any fasting needed for their primary procedure was coordinated with fasting for the OST. Some of these patients had lameness evaluations, diagnostic analgesia, and imaging procedures requiring standing sedation performed on the day of arrival, but at least 12 h of time elapsed between the conclusion of any procedures and the start of endocrine testing. For the field population, fasting was performed by owners or trainers under the guidance of the investigators and testing was performed by investigators first thing in the morning prior to horses being exercised.

Following the fasting period blood was collected via direct jugular venipuncture into red top and EDTA blood tubes. Horses were then given 0.15 mL/kg light corn syrup (Karo Light Corn Syrup) by mouth, and venipuncture was repeated at 60- and 90-min post-administration. The 0.15 mL/kg dose of light corn syrup was chosen over the 0.45 mL/kg dose as it is still the dose recommended by the Equine Endocrinology Group (EEG)^16^ and the volume of light corn syrup administered at the lower dose is more practical in a clinical setting. Horses were fed their normal diet following the conclusion of testing.

Insulin concentrations were measured using EDTA whole blood at the time of collection via a stall-side lateral flow assay (LFA, Well-ness Ready, Kentucky, USA), performed according to manufacturer’s instructions. The test cartridge and running buffer were warmed to room temperature prior to performing the test. Using the manufacturer’s bulb pipette, four drops of EDTA whole blood were added to the running buffer bottle. The bottle was inverted five times, and then four drops of the solution were applied to the sample window of the cartridge. The sample was allowed to flow fully across the test window prior to initiating the 15-min reading timer on the device.

Blood from the red top tubes was allowed to clot at room temperature for 30–60 min prior to centrifugation at 3000 × *g* for 15 min. Serum was transferred to cryovials and stored at −80°C until analysis. Serum insulin concentrations were measured using an automated fluorescence enzyme immunoassay (FEIA), the Tosoh AIA-360 (Tosoh Bioscience). Serum leptin concentrations were measured using a radioimmunoassay (Multispecies leptin RIA, Millipore). Both assays have been previously validated for use in horses.^22,23^ Serum total adiponectin serum concentrations were measured using a turbidimetric immunoassay (Adiponectin Turbidimetric Immunoassay Kit, ImmunoDiagnostics, Hong Kong) similar to that extensively used previously in ponies and horses.^24^ The assay was validated in the authors’ laboratory^25^: Mean (range) intra-assay coefficient of variation (CV%) was 2.3% (1.8%–2.8%); inter-assay, across-day CV% was 3.3% (3.2%–3.4%) and across reagent lot CV was 11.7% (9.0%–17.9%). Mean recovery upon dilution was 121.9% (75%–140.6%), and LLoQ 1.2 and 1.3 μg/mL using two methods.

Based on reference ranges provided by the laboratory performing the FEIA, a diagnosis of ID was made if the serum insulin concentration was >20 μIU/mL at T0, or >30 μIU/mL at T60 or T90 as measured by the FEIA. This cut-off is lower than the >45 μIU/mL recommended by the EEG.^16^ However, the diagnostic threshold recommended by the EEG is for samples measured via radioimmunoassay, and previous comparison data between the FEIA and radioimmunoassay show a constant bias of −10 to −20 μIU/mL, indicating that the FEIA consistently reads 10–20 μIU/mL lower than the radioimmunoassay.^23^

### 2.3 | Data analysis

A power analysis performed using the Scalex calculator with an estimated prevalence of 18% based on previous literature, a confidence level of 95%, and a desired precision of 5%–6% yielded a required sample size of between 158 and 227 horses.^26^ Data were assessed for normality using visual methods and Shapiro–Wilk tests. Normally distributed summary data were reported as mean and standard deviation, and non-normally distributed data were reported as median and range. Mann–Whitney *U* tests were used to assess differences in continuous variables between field and hospital populations, and Fisher Exact tests were used for categorical variables.

A multivariate mixed effects binary logistic regression was used to examine the relationships of the probability of ID diagnosis with the following independent variables: leptin/adiponectin ratio, sex, age, BCS, CNS, BCI, breed, and the interaction of the leptin/adiponectin ratio and sex. The model incorporated a random effect of individual horse to account for the lack of independence between repeated measures on the same animal. Estimated odds ratios and confidence intervals are reported alongside p values for each independent variable in the model.

Samples within the readable range of the stall-side assay (20–198 μIU/mL) were included in the assay agreement analysis (*N* = 400). A Pearson’s correlation coefficient was determined between the two assays. A Bland–Altman plot was created showing bias and 95% limits of agreement.

Receiver operator characteristic (ROC) curve analysis for evaluation of LFA accuracy for the OST was performed using data from all insulin samples (*N* = 540). Any samples measuring <20 μIU/mL by the LFA were inputted as 19 μIU/mL, and any samples measuring ≥199 μIU/mL were inputted as 199 μIU/mL. Any LFA measurement corresponding to a T0 FEIA measurement of >20 μIU/mL or a T60 or T90 FEIA measurement of >30 μIU/mL was entered as ID positive, and all other LFA measurements were entered as ID negative. Area under the curve (AUC) was calculated along with a 95% confidence interval (CI). ROC curve coordinates were used to determine possible cut-off values for the LFA to optimise sensitivity and specificity. The Youden index was calculated for each possible cut-off using the formula: *Sensitivity* + *Specificity* − 1.

ROC curve analysis for evaluation of the accuracy of fasted insulin concentrations measured by both the FEIA and LFA was performed using all T0 samples (*N* = 180). Any T0 insulin concentration from a patient who was classified as ID positive at any point during the OST was entered as ID positive, and all other T0 insulin concentrations were entered as ID negative. Analysis was performed separately for the LFA and FEIA using the same methods as above.

Statistical significance was set at *p* < 0.05 for all comparisons. Software used for statistical analysis included the base and stats packages in R v. 4.5.1 and GraphPad Prism v. 10.4.0.

## 3 | RESULTS

### 3.1 | Population demographics

A total of 180 horses and ponies were included in the study population with 140 evaluated in hospital and 40 evaluated in the field. For the hospital population, a total of 210 owners were offered study enrolment. Seventy of those owners declined enrolment or cancelled their appointments with the service. Enrolment was discontinued once 140 patients were tested. Of the 140 patients, 32 were tested on the day of arrival and had a median acclimation time of 3 h (range 3–6 h). The remaining 108 were tested during their scheduled hospitalisation with a median acclimation time of 18 h (range 12–72 h). The field horses were enrolled from five facilities specialising in different disciplines including dressage and driving ponies (*N* = 4), eventing (*N* = 6), hunter/jumpers (*N* = 8), saddleseat (*N* = 12), and reining (*N* = 10). Demographic data and morphometric parameters for the total study population, as well as the hospital and field subpopulations, are presented in Table 1. The distribution of breeds and disciplines differed significantly between field and hospital populations ( *p* < 0.001). There was no significant difference in the distribution of BCS or CNS between populations; however, mean BCI was significantly lower for field horses than hospital horses (7.64 ± 2.05 vs. 8.31 ± 1.84, *p* = 0.044). Mean weight was also significantly lower for field horses (496 ± 104 kg vs. 560 ± 78 kg, *p* < 0.001). In the overall population, 109/180 horses (60.5%) were overweight with a BCS of ≥6. Visible crest fat (CNS ≥ 2) was present in 100/180 (55.6%). Owners reported a history of veterinarian-diagnosed PPID in 4/180 (2.2%) cases. All four horses were in the hospital population, and all were receiving pergolide.

### 3.2 | Endocrine testing

Leptin concentrations were increased (>10 ng/mL) in 44/180 horses (24.4%), and adiponectin concentrations were decreased (<8 μg/mL) in 100/180 (55.6%). Hormone concentration data for the total study population, as well as the hospital and field subpopulations, are presented in Table 1. Median leptin and adiponectin concentrations did not differ between field and hospital populations. Median T0 insulin was significantly lower in field horses than hospital horses (4.5, 1.5–27.3 vs. 7.1, 0.9–48, *p* < 0.001), but both were within the normal range for fasting insulin concentrations, and median T60 and T90 insulin concentrations did not differ.

Insulin dysregulation was diagnosed in 41/180 horses (22.8%, 95% CI 17.0%–29.6%). Of the 41 ID horses, 11 (26.8%) had increased fasted (T0) insulin. All horses with increased T0 insulin concentrations also had increased T60 and T90 insulin concentrations. The distribution of positive and negative horses at each time point for the OST is displayed in Figure 1. Summary data for ID versus non-ID horses are displayed in Table 2. Statistical comparisons between ID and non-ID populations were not made to avoid duplication of analysis with the regression model. Of the four horses with owner-reported PPID, two were diagnosed with ID. Due to the small number of horses with PPID, formal statistical comparisons were not made regarding the prevalence of ID in this subpopulation. A total of 53/180 (29.4%) horses were reported to be on systemic medications at time of study enrolment. Five of these horses were in the field population, and the remaining 48 were in the hospital population. Fifteen different medications were reported, and due to the small number of horses receiving each medication, no formal statistical comparisons were made. Systemic medication data is available in Table S1.

### 3.3 | Variables associated with ID

The results of the mixed effects binary logistic regression model are given in Table 3. There was a statistically significant interaction of leptin/adiponectin ratio and sex (odd ratio [OR] for female vs. male = 0.920, 95% CI 0.854–0.989), indicating that the relationship of the leptin/adiponectin ratio to the probability of ID diagnosis differs depending on sex. Additional calculations showed that for male horses, there was a statistically significant relationship between leptin/adiponectin ratio and the probability of ID diagnosis (OR = 1.102, 95% CI 1.036–1.171; χ^2^(1) = 9.59, *p* = 0.002). In male horses, for each 0.1 unit increase in the leptin/adiponectin ratio, odds of ID increased by 10.2%. There was not a statistically significant relationship for female horses (OR = 1.013, 95% CI 0.975–1.053; χ^2^(1) = 0.43, *p* = 0.513). There were no other statistically significant relationships with the probability of ID diagnosis.

### 3.4 | Assay agreement

A total of 540 samples were measured by both assays. Of these samples, 140 were outside of the readable range of the LFA, with 138 measuring <20 μIU/mL and 2 measuring ≥199 μIU/mL. All 138 samples measuring <20 μIU/mL were also measured to be <20 μIU/mL with the FEIA. The two samples measuring ≥199 μIU/mL were above the diagnostic threshold for ID with the FEIA but measured substantially lower than the LFA (57.2 and 65.6 μIU/mL).

The 400 samples measuring between 20 and 198 μIU/mL were included in assay agreement analysis. Pearson’s correlation coefficient showed a strong positive correlation between assays with an r of 0.81 ( *p* < 0.001, 95% CI 0.78–0.84). The Bland–Altman plot is displayed in Figure 2. Bias (LFA – FEIA) was 13.62 ± 9.89 μIU/mL, and 95% limits of agreement were −5.75 to 32.99 μIU/mL.

The ROC curve for classification of ID status based on LFA-measured insulin concentration is shown in Figure 3. All 540 samples were included in the analysis, with samples reading <20 inputted as 19 μIU/mL and those ≥199 inputted as 199 μIU/mL. Accuracy of the LFA for ID classification relative to the FEIA diagnosis was excellent (AUC = 0.95, 95% CI 0.93–0.97). The optimal cut-off value for LFA insulin based on the Youden index was >36.9 μIU/mL, providing a sensitivity of 84.5% (95% CI 74.4%–91.1%) and a specificity of 90.9% (95% CI 87.9%–93.1%). At this threshold, negative and positive predictive values in the study population were 95.4% and 72.4% respectively. [Correction added on 25 August 2026, after first online publication: In the preceding sentence, ‘90.2%’ was corrected to ‘72.4%’.] A lower threshold of >30.7 μIU/mL can be used to improve sensitivity (97.2%, 95% CI 90.3%–99.5%) and negative predictive value (99.0%) with some sacrifice in specificity (77.4%, 95% CI 73.4%–81.0%) and positive predictive value (55.1%). [Correction added on 25 August 2026, after first online publication: In the preceding sentence, ‘81.1%’ was corrected to ‘55.1%’.]

### 3.5 | Diagnostic cut-offs for fasting insulin

To determine if superior cut-offs could be identified to improve the sensitivity and specificity of fasted insulin concentrations, ROC curves were created using only the fasted insulin concentrations (*N* = 180) as measured by the FEIA (Figure 4A) and LFA (Figure 4B). Fasted samples measured by the FEIA had good accuracy for ID classification (AUC = 0.89, 95% CI 0.83–0.95). The optimal cut-off value based on the Youden index was >10.1 μIU/mL, providing a sensitivity of 73.2% (95% CI 58.1%–84.3%) and a specificity of 90.7% (95% CI 84.7%–94.5%). At this threshold, negative and positive predictive values in the study population were 92.3% and 70.5% respectively. [Correction added on 25 August 2026, after first online publication: In the preceding sentence, ‘88.7% and 92.3%’ was corrected to ‘92.3% and 70.5%’ respectively.] Fasted samples measured by the LFA had fair accuracy for ID classification (AUC = 0.71, 95% CI 0.61–0.81). The optimal cut-off value based on the Youden index was >21.8 μIU/mL, providing a sensitivity of 68.3% (95% CI 53.0%–80.4%) and a specificity of 69.8% (95% CI 61.7%–76.8%). At this threshold, negative and positive predictive values in the study population were 88.6% and 40.8% respectively. [Correction added on 25 August 2026, after first online publication: In the preceding sentence, ‘69.3%’ was corrected to ‘40.8%’.]

## 4 | DISCUSSION

In this population of sport horses, the prevalence of ID was 22.8%, highlighting that ID can be a comorbidity in a significant proportion of equine athletes. This study adds to the body of literature regarding variables that may help practitioners identify horses at greatest risk for ID and provides further validation of a stall-side insulin assay to improve the practicality of diagnosis and monitoring in a field setting.

The true prevalence of ID in this population is estimated to be between 17.0% and 29.7%. Given this range of values, we cannot accept our hypothesis that the prevalence in this population would be higher than the 18.3% previously reported by Pleasant et al. This hypothesis was based on the fact that Pleasant et al. used baseline insulin concentrations for ID diagnosis, while our study used the more sensitive OST.^10^ Although the two study populations are similarly diverse in terms of breed and body condition distribution, over half of the Pleasant et al. study population was comprised of horses performing no exercise.^10^ Given that exercise has been shown to improve insulin dynamics,^27^ the difference in athletic use for the horses included in these two populations may explain the lack of difference in prevalence despite the use of a more sensitive diagnostic in the present study. The prevalence reported here is higher than that of 4.8% reported by Heaton et al.^11^ The population in the Heaton study was tested using an OST but was composed solely of non-obese stock-type horses.^11^ Given that the majority of the population described here was overweight, it is unsurprising that the prevalence of ID was higher in our population.

To address concerns that a referral population may include a higher proportion of horses out of work related to their injuries or include horses more likely to have comorbidities along with primary orthopaedic disease, a population of healthy horses tested in the field was included. Although the field population included a different breed and discipline distribution than the hospital population, there were no differences in BCS, CNS, adiponectin, leptin, or overall prevalence of ID between the two populations. The field population had lower mean BCI and weight, likely due to the inclusion of a greater proportion of ponies. There was a statistically significant difference between median baseline insulin concentrations for the two subpopulations, but the difference between an insulin of 4.5 and 7.1 μIU/mL is not considered clinically significant. Despite differences in breed and discipline representation, the similarities in hormone concentrations and prevalence of ID positive horses demonstrate that the 140 horses tested inhospital are still representative of the broader sport horse population.

Of the 41 horses diagnosed with ID in this population, only 26.8% had fasted insulin concentrations above the reference laboratory’s diagnostic threshold for ID. Previous literature has shown that fasted insulin concentrations fail to correctly diagnose truly positive horses in 85% of cases when compared to the combined glucose-insulin test.^8^ ROC curve analysis was performed for fasted samples measured using both the FEIA and LFA to determine if different diagnostic cut-offs could improve diagnostic utility, as the T0 diagnostic threshold provided by the reference laboratory performing the FEIA was created for both fasted and hay/pasture-fed baseline samples. The optimal cut-off for the FEIA was determined to be >10.1 μIU/mL, achieving a sensitivity of 73.2% and a specificity of 90.7%. At this threshold, 27/41 (65.9%) ID horses would be identified using the fasted sample. However, 13 false-positive results would also occur. The optimal cut-off for the LFA, >21.8 μIU/mL, achieves a similar sensitivity at 68.3%, but a lower specificity at 69.8%. The LFA would correctly identify 26/41 (63.4%) ID horses at this threshold but would also yield 40 false-positive results. These lower thresholds may be useful as an initial screening test or in cases where dynamic testing is not feasible for financial or patient behaviour reasons but should not replace dynamic testing altogether. Samples obtained in a baseline fed state (hay/pasture, no grain) appear to have improved accuracy over fasted samples, but further work is needed to determine diagnostic thresholds for each insulin assay.^2^

To provide practitioners with information that can be used to guide case selection for dynamic insulin testing, multivariate analysis was performed to identify predictors of ID. Although historically considered a disease of overweight and obese horses, neither BCS nor CNS were significant predictors of ID risk, indicating that practitioners cannot rely on the appearance of the horse for clinical decision making. This differs from previous reports in other populations showing correlations between BCS or CNS and insulin concentration or ID diagnosis.^10,28–30^ The high proportion of overweight horses in this population may have made it more challenging to discern between overweight, metabolically normal horses and overweight, metabolically abnormal horses. The BCI, a more objective assessment of body morphometry originally described by Potter et al.,^21^ was also not a significant predictor of ID. However, the authors found it to be a useful tool for objective follow-up monitoring of weight loss in overweight horses after study enrolment. This study additionally found no specific breed association with ID risk, but several breeds classically considered at high risk for ID such as pony breeds, Arabians, and Morgans^31–33^ were absent from this population or present in small numbers. Therefore, despite the large number of horses in the overall population, the presence of subgroups with small numbers limited statistical power to detect differences in ID risk for these groups.

The only significant predictor of ID risk in this population was the leptin/adiponectin ratio, and this predictor was significant for male horses only. In human patients and in horses, leptin concentrations tend to increase with increasing fat mass.^10,34,35^ Adiponectin concentrations decrease with increasing fat mass, and low adiponectin concentrations have been identified as predictors of complications of obesity in both species.^1,2,36^ The ratio of these two adipokine concentrations has been shown to be more strongly correlated with insulin resistance in human patients, and is a better predictor of complications of obesity than either hormone alone.^37,38^ Horses with ID and horses with laminitis have been shown to have higher leptin/adiponectin ratios than non-ID and non-laminitic horses.^18^ It is possible that this ratio better reflects the amount of dysfunctional fat a patient has relative to their overall fat mass, as those with lower adiponectin concentrations will have higher ratios. In our population, each 0.1-point increase in the ratio was associated with 10% increased odds of ID in male patients. The ratio was not a significant predictor for female patients. Differential predictive value of adipokines based on sex has not been previously reported in horses, but has been reported in humans.^39,40^ Human males are reported to have higher leptin and adiponectin values on average than females, thought to be related to effects of oestrogen and testosterone and reflective of different fat deposition patterns across sexes.^41,42^ Further research is needed to determine if this differential predictive value of adipokines holds true in other equine populations and to determine potential mechanisms underlying these differences.

The Wellness Ready stall-side LFA can provide quantitative insulin results from fresh EDTA whole blood samples in 15 min, allowing practitioners to evaluate insulin concentrations and make same-day management decisions. There is one previous study showing good agreement (*r* = 0.90) between the Wellness Ready and a radioimmunoassay in a laboratory setting using 99 samples with insulin concentrations between 20 and 100 μIU/mL.^17^ The agreement shown here between the Wellness Ready and Tosoh immunoassay was lower (*r* = 0.81), and a fan-shaped distribution of data points was visible in the Bland–Altman plot, indicating greater variability at higher insulin concentrations. A similar data trend was reported in the original validation study, and the effect of this variability was likely magnified in our population due to the inclusion of samples with insulin concentrations >100 μIU/mL. Occasional spuriously high and low measurements were seen as evidenced by the points outside of the 95% limits of agreement lines, as well as the two samples measuring ≥199 μIU/mL with the LFA but substantially lower with the FEIA. It has been previously proposed that spuriously high results seen with other equine insulin assays may be due to the presence of heterophilic antibodies that are recognised only by specific assays, or through the binding of related hormones or hormone-binding proteins.^23^ Spuriously low results have not been reported with other equine insulin assays, but have also been reported in relation to heterophilic antibodies in humans.^43^ Further research is needed to better understand the role of these interferents in equine insulin assays, and practitioners should consider repeat insulin measurements using a different assay if an initial result does not match the clinical impression.

There remains no true gold standard tool for the diagnosis of ID. The FEIA was selected as a reference assay for this study due to its accuracy at a wide range of insulin concentrations and practicality for use in a clinical setting.^23^ The mean bias reported from the BlandAltman plot indicates that on average the LFA reads 13.62 μIU/mL higher than the FEIA. The reported mean bias for the FEIA relative to the radioimmunoassay is −16.7 μIU/mL.^23^ These bias values emphasise the need for practitioners to understand that insulin results may not be directly comparable across assays, and the importance of assay-specific reference ranges. The Youden index is useful for establishing a diagnostic cut-off that maximises overall test performance, but does not take into consideration disease prevalence nor the relative importance of sensitivity and specificity for a given test.^44^ The LFA is most useful as a screening tool in the field to rapidly determine whether a patient might be at risk for ID and related complications such as laminitis. Because of this, a false negative result would be more detrimental than a false positive result, and prioritising test sensitivity is beneficial. Utilising a lower cut-off value of >30.7 μIU/mL achieves excellent sensitivity (97.2%) while maintaining adequate specificity (77.4%). The cut-off value proposed here should be evaluated further in different horse populations to confirm validity.

It is important to note that the OST is also not a perfect diagnostic test, and some controversy exists regarding execution and interpretation of OST results. The authors chose to use the lower 0.15 mL/kg dose of light corn syrup here rather than the more recently described 0.45 mL/kg dose.^45^ The 0.45 mL/kg has been shown to cause significantly higher peak insulin concentrations, and unlike the 0.15 mL/kg dose, was able to differentiate ponies with and without prior history of laminitis.^45^ However, the two doses have not been directly compared in regards to their ability to differentiate ID and non-ID horses. It is reasonable to assume that the higher dose may be more sensitive, but it is possible that the increased sensitivity comes at the sacrifice of specificity. Additionally, in the authors’ experience, the volume of light corn syrup required for the 0.45 mL/kg dose is challenging to practically administer via oral dosing. For these reasons, as well as the fact that the 0.15 mL/kg dose is still recommended by the EEG, the authors chose to use the lower dose in this study. Response to the OST has been shown to be variable over time in individual horses, and in some cases can vary enough to change ID classification.^46,47^ Therefore, it is possible that some horses were misclassified by the OST, and pairing the OST with another dynamic insulin test could have improved accuracy. Previous studies have shown conflicting results as to the impact of season on ID status classification.^47–49^ Due to the nature of this study, it was not feasible to restrict testing to a particular season of the year, and thus it is possible that some horses may have been classified differently if tested in a different season. There is also some evidence that breed-specific diagnostic thresholds would improve the accuracy of the OST and other measures of metabolic health,^50^ but this practice has not been widely adopted and further research is needed to establish these thresholds.

This study had several limitations, the first of which was that enrolment was voluntary, meaning that owners who had greater concern about ID in their horses may have been more likely to enrol, overestimating the true prevalence of ID in the population. Secondarily, there is some evidence that transport stress can falsely elevate post-OST insulin concentrations.^51^ The majority of horses tested inhospital were allowed to assimilate to the hospital environment overnight (108/140), but it is possible that horses tested on the day of arrival (32/140) had altered insulin responses due to stress. However, the field population in which horses were tested in their home environment had a similar prevalence of ID, indicating that the number of false positive results related to transportation stress is likely low. Finally, this study was performed in a single geographic area and may not represent the ID prevalence in horses outside of the southeastern portion of the United States where other breeds and disciplines may predominate. The ROC analysis performed determined diagnostic thresholds using the entire data set rather than determining thresholds using a subpopulation and testing those thresholds on the remainder of the population. Although this data set is large compared to some previous studies, the splitting of the population into two groups for threshold selection and testing would result in the use of a small subpopulation that may not be representative of the overall population. Therefore, it is important to note that the diagnostic thresholds provided here require validation in an outside population to determine their broader applicability.

In conclusion, this study demonstrates that 23% of patients in a population of sport horses of various breeds and disciplines were diagnosed with ID. Additionally, 74% of cases required an OST for diagnosis, highlighting the importance of dynamic insulin testing for accurate diagnosis. No morphometric parameters were associated with ID risk, indicating that practitioners cannot rely on patient appearance to predict ID status. The leptin/adiponectin ratio may be a useful baseline predictor of ID risk in males but is not a significant predictor in females. The Wellness Ready stall-side insulin assay shows overall good agreement with the validated Tosoh immunoassay, achieving a negative predictive value of 99.0% and a positive predictive value of 55.1% at a proposed cut-off of >30.7 μIU/mL. [Correction added on 25 August 2026, after first online publication: In the preceding sentence, ‘81.1%’ was corrected to ‘55.1%’.] However, occasional spuriously high and low readings were seen which may complicate interpretation of results. Future studies should seek to understand the cause for these spurious results and evaluate the diagnostic cut-offs proposed here in different populations. Additionally, there is a need for future large-scale, multicentre prevalence studies and prospective studies evaluating laminitis risk over time in sport horse populations. This study underscores the importance of ID in our equine athletes and provides practitioners with valuable information regarding the importance of dynamic insulin testing and case selection.

## Supplementary Material

Supplemental 1

SUPPORTING INFORMATION

Additional supporting information can be found online in the Supporting Information section at the end of this article.

## Figures and Tables

**FIGURE 1:**
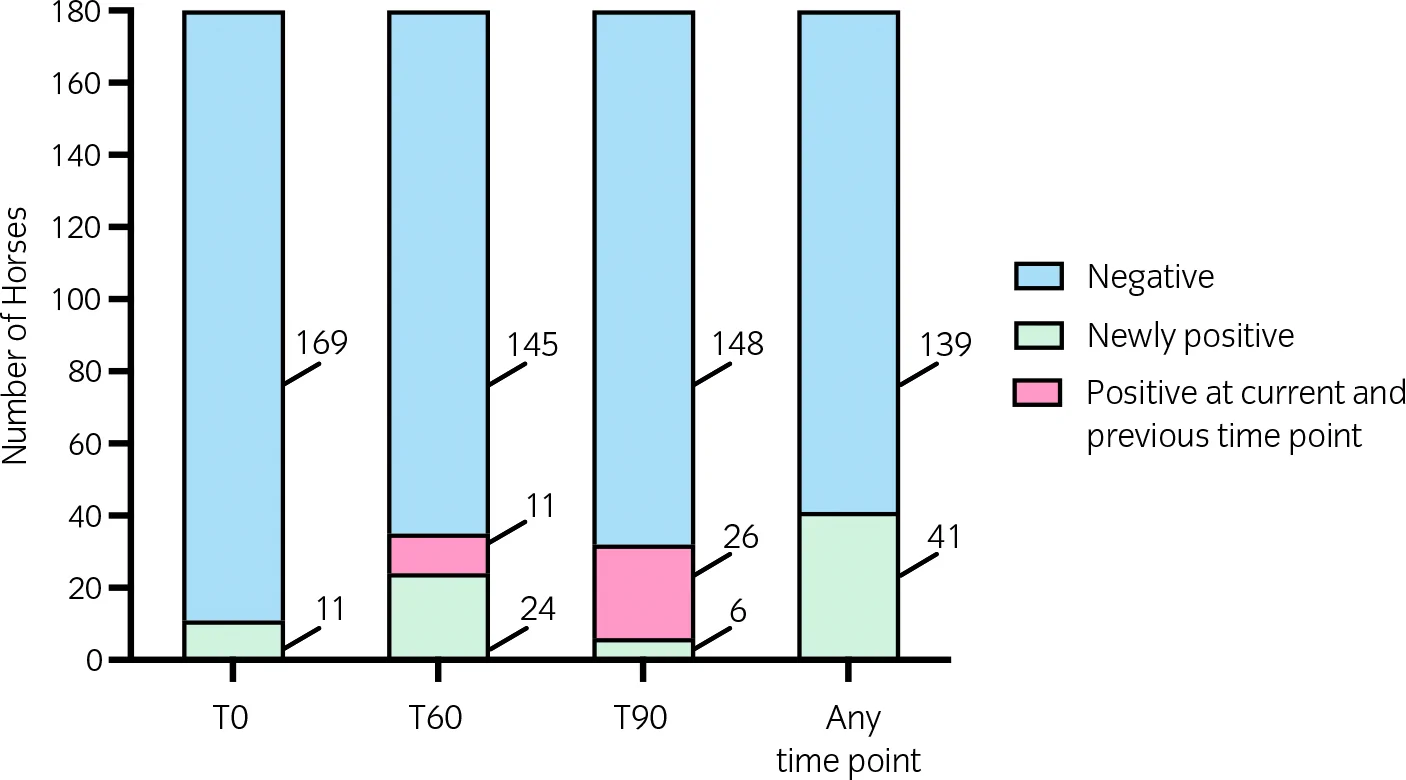
Oral sugar test results for *N* = 180 horses. Positive diagnosis for insulin dysregulation (ID) defined as fasted insulin (T0) measuring >20 μIU/mL or 60- or 90-min post-Karo syrup (T60 or T90) insulin measuring >30 μIU/mL on fluorescence enzyme immunoassay. Blue denotes horses negative for ID, green denotes horses newly identified as positive at each time point, and pink denotes horses positive at the current time point and at a previous time point. Data reported as number of horses in each category.

**FIGURE 2:**
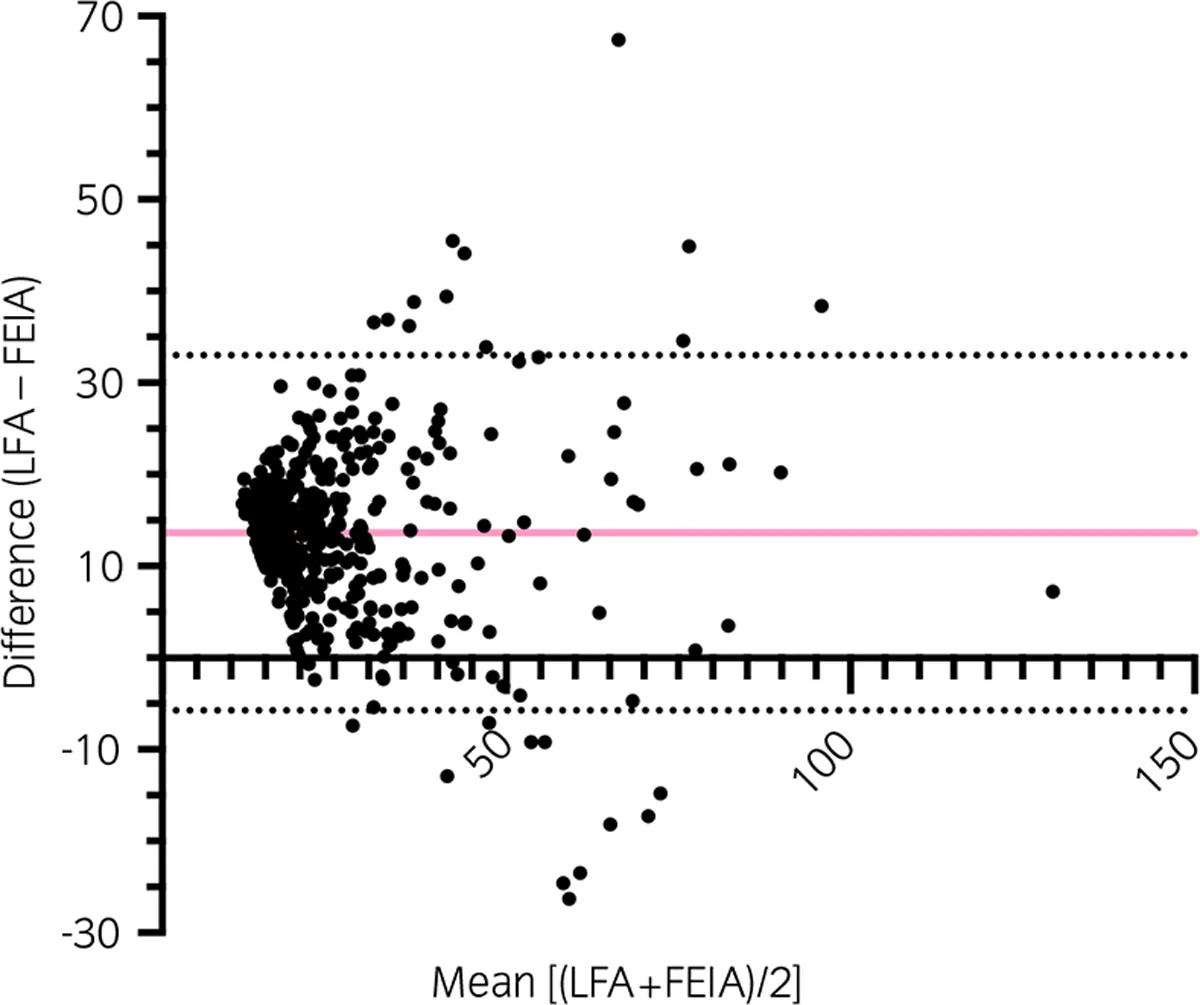
Bland–Altman plot of the absolute difference between equine insulin concentrations (*N* = 400) measured via fluorescence enzyme immunoassay (FEIA) or lateral flow assay (LFA). Black dotted lines denote 95% limits of agreement (−5.75 to 32.99 μIU/mL). Mean bias (13.62 μIU/mL) denoted by solid pink line.

**FIGURE 3:**
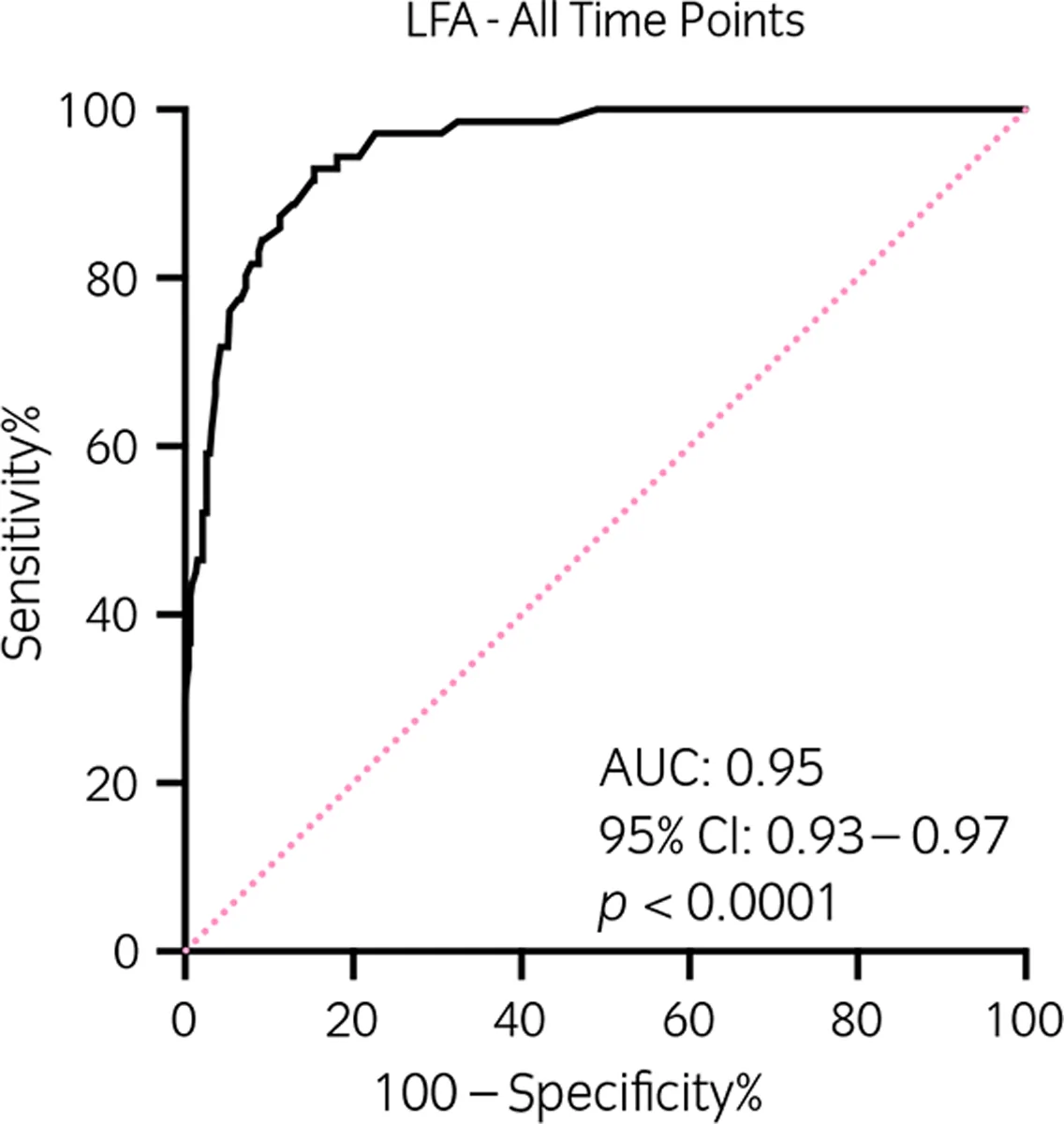
Receiver operator characteristic curve for classification of insulin dysregulation status by insulin concentrations measured by the stall-side lateral flow assay (LFA). Dotted pink line denotes an uninformative test for which the area under the curve (AUC) is 0.5. Solid black line denotes actual test performance.

**FIGURE 4:**
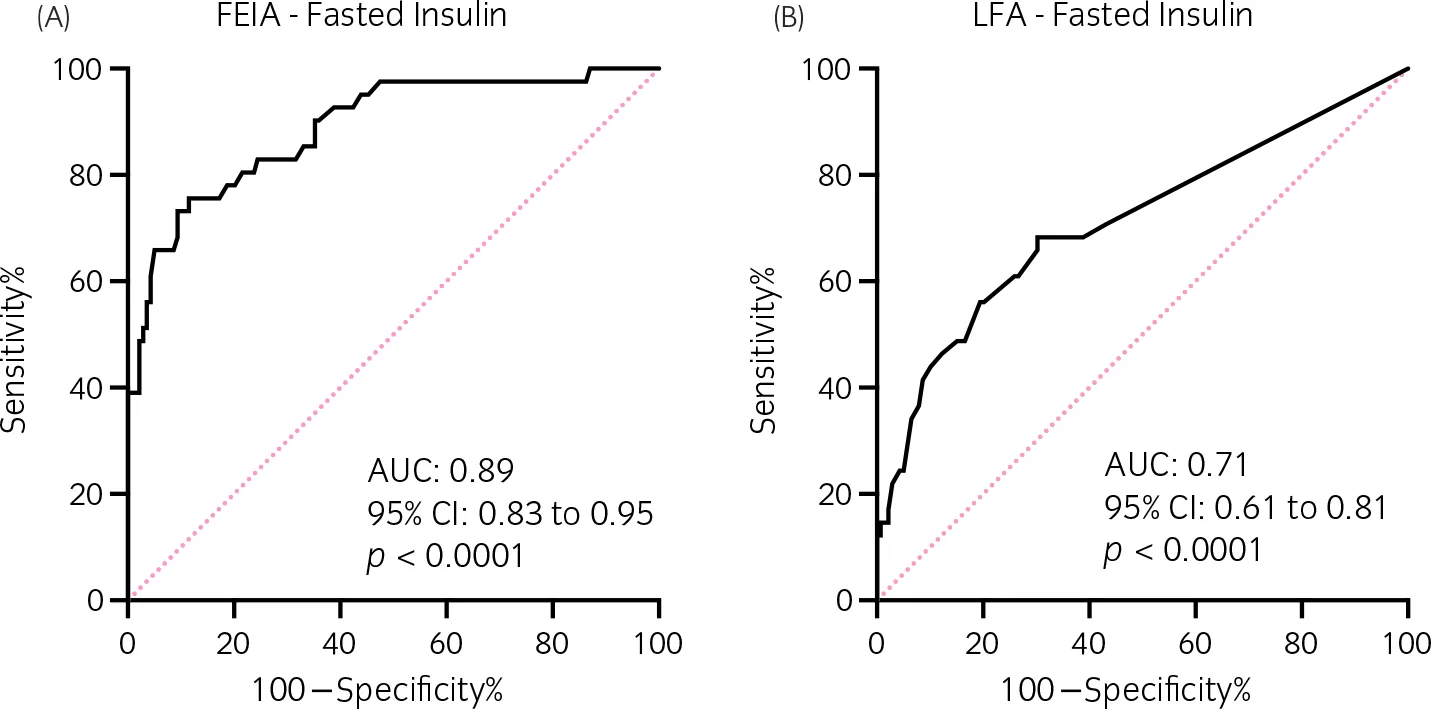
Receiver operator characteristic curves for classification of insulin dysregulation status by fasted insulin concentrations measured by the fluorescence enzyme immunoassay (FEIA, A) and stall-side lateral flow assay (LFA, B). Dotted pink line denotes an uninformative test for which the area under the curve (AUC) is 0.5. Solid black line denotes actual test performance.

**TABLE 1:** Demographic data for the overall study population (*N* = 180) and the hospital (*N* = 140) and field (*N* = 40) subpopulations.

	Total population (*N* = 180)		Hospital population (*N* = 140)		Field population (*N* = 40)		
	Categorical variables						
	*n*	% of *N*	*n*	% of *N*	*n*	% of *N*	*p*

Sex							
Mares	58	32.2%	43	30.7%	15	37.5%	0.446
Geldings	121	67.2%	97	69.3%	24	60.0%	
Stallions	1	0.6%	0	0%	1	2.5%	
Breed							
Warmblood	71	39.4%	62	44.3%	9	22.5%	0.001*
QH/Paint	44	24.4%	33	23.6%	11	27.5%	
Thoroughbred	22	12.2%	19	13.6%	3	7.5%	
Pony	15	8.3%	7	5.0%	8	20.0%	
Saddlebred	13	7.2%	4	2.9%	9	22.5%	
Arabian	5	2.8%	5	3.6%	0	0%	
Other	10	5.6%	10	7.1%	0	0%	
Discipline							
Hunter/jumper	52	28.9%	44	31.4%	8	20.0%	<0.001*
Dressage	46	25.6%	43	30.7%	3	7.5%	
Western	35	19.4%	25	17.9%	10	25.0%	
Eventing	18	10.0%	12	8.6%	6	15.0%	
Saddleseat	15	8.3%	3	2.1%	12	30.0%	
Trail	10	5.6%	10	7.1%	0	0%	
Other	4	2.2%	3	2.1%	1	2.5%	

	Total population (*N* = 180)		Hospital population (*N* = 140)		Field population (*N* = 40)		
	Continuous variables						
	Mean (SD) or median	Range	Mean (SD) or median	Range	Mean (SD) or median	Range	*p*

Age	10	2–23	10	2–23	10	2–22	0.979
Weight	546 (88)	240–768	560 (78)	258–765	496 (104)	240–768	<0.001*
BCS	6	4–9	6	4–9	6	4–7	0.728
CNS	2	0–4	2	0–4	2	0–3	0.896
BCI	8.16 (1.91)	3.72–12.97	8.31 (1.84)	3.72–12.97	7.64 (2.05)	4.34–12.02	0.044*
Leptin	7.07	0.9–47.81	7.01	0.9–33.1	7.46	1.94–47.81	0.940
Adiponectin	7.7	2.5–16.6	7.8	3–16.6	7.1	2.5–14	0.250
Leptin/adipo ratio	0.904	0.125–11.119	0.897	0.125–5.446	0.959	0.294–11.119	0.588
T0 insulin	6.8	0.9–48	7.1	0.9–48	4.5	1.5–27.3	<0.001*
T60 insulin	16.7	3.4–79.8	16.6	3.9–79.8	17	3.4–58.2	0.978
T90 insulin	14.3	2.5–125.8	14.3	2.5–80.5	13.9	4.2–125.8	0.570

*Note*: Data for categorical variables are presented as number of horses and percent of population, and comparisons between groups are made using Fisher Exact tests. Data for continuous variables are presented as median (SD) or median and range, and comparisons between groups are made using Mann–Whitney *U* tests with significance set at *p* < 0.05, and significant values are denoted with an asterisk.

**TABLE 2:** Demographic data for horses with (*N* = 41) and without (*N* = 139) insulin dysregulation (ID).

	Non-ID (*N* = 139)		ID (*N* = 41)	
	Categorical variables			
	*n*	% of *N*	*n*	% of *N*

Sex				
Mares	41	29.5%	17	30.7%
Geldings	97	69.8%	24	69.3%
Stallions	1	0.7%	0	0%
Breed				
Warmblood	57	41.0%	14	34.1%
QH/Paint	31	22.3%	13	31.7%
Thoroughbred	20	14.4%	2	4.9%
Pony	10	7.2%	5	12.2%
Saddlebred	10	7.2%	3	7.3%
Arabian	5	3.6%	0	0%
Other	6	4.3%	4	9.8%
Discipline				
Hunter/jumper	47	33.8%	5	12.2%
Dressage	33	23.7%	13	31.7%
Western	25	18.0%	10	24.4%
Eventing	13	9.4%	5	12.2%
Saddleseat	12	8.6%	3	7.3%
Trail	7	5.0%	3	7.3%
Other	2	1.5%	2	4.9%

	Non-ID (*N* = 139)		ID (*N* = 41)	
	Continuous variables			
	Mean (SD) or median	Range	Mean (SD) or median	Range

Age	10	3–23	11	2–23
Weight	547 (88)	240–768	542 (88)	340–765
BCS	6	4–8	6	4–9
CNS	2	0–4	2	0–4
BCI	7.89 (1.81)	3.72–12.70	9.07 (1.98)	5.21–12.97
Leptin	6.38	0.9–47.81	11.82	3.11–26.7
Adiponectin	7.8	2.7–16.6	7.2	2.5–14.5
Leptin/adiponectin ratio	0.797	0.125–11.119	1.800	0.247–5.446
T0 insulin	5.7	0.9–16.7	14	3.1–48
T60 insulin	14.2	3.4–29.8	42.1	18.4–79.8
T90 insulin	12.4	2.5–30	39.2	9.6–125.8

*Note*: Data for categorical variables are presented as number of horses and percent of population. Data for continuous variables are presented as median (SD) or median and range.

**TABLE 3:** Binary logistic regression for variables associated with insulin dysregulation.

Variable	OR	95% LCL	95% UCL	χ^2^	df	*p*

Sex				2.28	1	0.131
Male	Reference					
Female	1.947	0.817	4.684			
BCS				1.64	1	0.200
<6	Reference					
≥6	1.422	0.833	2.464			
CNS						
<2	Reference					
≥2	1.152	0.620	2.128	0.21	1	0.651
Breed				1.12	5	0.952
Warmblood	Reference					
Pony	1.673	0.383	6.687			
QH/Paint	1.351	0.435	4.198			
Saddlebred	1.501	0.266	7.022			
Thoroughbred	0.777	0.103	3.865			
Other	1.404	0.266	6.594			
Age	1.075	0.981	1.179	2.43	1	0.119
BCI	1.115	0.828	1.510	0.51	1	0.474
Leptin/adiponectin ratio	1.102	1.038	1.175	8.78	1	0.003*
Leptin/adiponectin ratio × Sex (female vs. male)	0.920	0.854	0.989	5.20	1	0.023*

*Note*: Individual horse was included as a random effect to account for the lack of independence between repeated measures on the same animal. Estimated odds ratios (OR) and confidence intervals (95% CI) are reported alongside *p* values for each independent variable in the model. Asterisk denotes statistical significance of *p* < 0.05.

## Data Availability

The data that support the findings of this study are openly available in Dryad at https://doi.org/10.5061/dryad.fbg79cpbb.

## DATA INTEGRITY STATEMENT

Kimberly L. Hallowell and Lauren V. Schnabel had full access to all the data in the study and take responsibility for the integrity of the data and the accuracy of the data analysis.
